# Sickle Cell Disease and *Bartonella* Spp. Infection

**DOI:** 10.4084/MJHID.2012.046

**Published:** 2012-06-30

**Authors:** Paulo Eduardo Neves Ferreira Velho, Marna Elise Ericson, David Mair, Kalpna Gupta

**Affiliations:** 1 Division of Dermatology, Department of Medicine, University of Campinas/UNICAMP, Brazil and Research Associate of the Department of Dermatology, University of Minnesota, USA; 2 Department of Dermatology/Center for Drug Design, University of Minnesota, USA; 3 American Red-Cross-North Central Region, USA; 4 Division of Hematology, Oncology and Transplantation, Department of Medicine, University of Minnesota, USA

**To the Editor,**

We read with great interest the recent article *The role of infection in the pathogenesis of vaso-occlusive crisis in patients with sickle cell diseases,* written by Dr. Ahmed SG. He pointed that these patients have impaired immunity and are thus predispose to infections which can precipitate the painful crisis.^1^ SCD is the most common hereditary hematologic disorder in the world and remains a significant global health problem with high relevance to low- and middle-income countries.^2^ The vast majority of SCD patients live in underdeveloped nations with high prevalence and transmission rates of infections.^1^ In equatorial Africa 10–40% of native populations have sickle (S) gene.^2^ In Brazil some afro-descendent groups have a prevalence of up to 10% of S gene and the disease is a relevant public health problem.^2^,^3^ SCD patients have infections which are often asymptomatic.^1^ Gram-negative infection are frequent in pneumococcal vacccineted SCD patients that had been splenectomized and functional asplenia is as frequent as 90% by 6 years of age.^4^

These patients frequently need blood transfusion and transmission of pathogens via transfusion in SCD patient infection has been documented.^5^ Amongst the potential gram-negative infections *Bartonella* spp. are emergent bacteria with worldwide distribution. An increasing number of *Bartonella* spp. are regarded as zoonotic pathogens, creating a public health concern for human and veterinary medicine. The extent of *Bartonella* spp. infection is underestimated.^6^ *Bartonella* spp. bacteremia is potentially fatal, especially in immunodeficient patients. Immunocompetent individuals are also at risk for chronic infection by this intra-erythrocyte and intra-endothelial agent though the infection can be asymptomatic.^7^ A broad spectrum of clinical manifestations have been related to *Bartonella* spp. infection, many of which were considered idiopathic prior to the diagnosis of chronic *Bartonella* spp. infection. A recent study from the United States of America found that almost 50% of patients with non-specific symptoms (fatigue, sleeplessness, joint and muscle pain etc.) had positive *Bartonella* spp. serology and/or blood PCR positive to *Bartonella* spp. One in four patients had *Bartonella* spp. bacteremia.^8^

Previous studies show that blood donors can have asymptomatic bacteremia.^7^ There are no gold-standard tests to confirm *Bartonella* spp. infection and false-negative results are frequent even with serology and multi-step molecular and microbiological techniques.^8^ Thus diagnostic tests for *Bartonella* spp. remains challenging, warranting development of more sensitive and reproducible diagnostic methods.

It is likely that SCD patients could have a higher prevalence of *Bartonella* spp. infection rate as they present with inflammation, endothelial activation, asplenia, and the need for frequent blood transfusions; pathological features that can promote the invasion and progression of *Bartonella* spp..^1^,^4^,^6^ Pain, fatigue and fever, characteristic features of *Bartonella* spp. infection are manifest in SCD. Therefore, coordinated international efforts should be initiated to evaluate the relevance of this infection in SCD and other chronic immunodeficient patients.
